# Dental Pulp Stem Cells Derived From Adult Human Third Molar Tooth: A Brief Review

**DOI:** 10.3389/fcell.2021.717624

**Published:** 2021-10-12

**Authors:** Ashraf Al Madhoun, Sardar Sindhu, Dania Haddad, Maher Atari, Rasheed Ahmad, Fahd Al-Mulla

**Affiliations:** ^1^Department of Genetics and Bioinformatics, Dasman Diabetes Institute, Dasman, Kuwait; ^2^Department of Animal and Imaging Core Facilities, Dasman Diabetes Institute, Dasman, Kuwait; ^3^Department of Immunology and Microbiology, Dasman Diabetes Institute, Dasman, Kuwait; ^4^Biointelligence Technology Systems S.L., Barcelona, Spain

**Keywords:** dental pulp stem cells, surface markers, heterogeneity, immunomodulation, hepatogenic and pancreatic differentiation, human DPSCs

## Abstract

The fields of regenerative medicine and stem cell-based tissue engineering have the potential of treating numerous tissue and organ defects. The use of adult stem cells is of particular interest when it comes to dynamic applications in translational medicine. Recently, dental pulp stem cells (DPSCs) have been traced in third molars of adult humans. DPSCs have been isolated and characterized by several groups. DPSCs have promising characteristics including self-renewal capacity, rapid proliferation, colony formation, multi-lineage differentiation, and pluripotent gene expression profile. Nevertheless, genotypic, and phenotypic heterogeneities have been reported for DPSCs subpopulations which may influence their therapeutic potentials. The underlying causes of DPSCs’ heterogeneity remain poorly understood; however, their heterogeneity emerges as a consequence of an interplay between intrinsic and extrinsic cellular factors. The main objective of the manuscript is to review the current literature related to the human DPSCs derived from the third molar, with a focus on their physiological properties, isolation procedures, culture conditions, self-renewal, proliferation, lineage differentiation capacities and their prospective advances use in pre-clinical and clinical applications.

## Introduction

Dental pulp stem cells (DPSCs) are a unique population of cells embedded within the pulp cavity of the impacted third molars. DPSCs were initially isolated and characterized by Gronthos et al. (2000). Subsequently, several investigators have reported DPSCs’ isolation, characterization, differentiation, and banking (Atari et al., 2012; Ferro et al., 2012a; Tirino et al., 2012). In comparison to other adult stem cells, DPSCs are noted for their high recovery rate from the disposable dental pulp after occlusion management. Their isolation procedure involves non-invasive techniques and has no notable ethical constraints. Significantly, DPSCs’ stemness, viability, proliferation, and differentiating capabilities are not compromised after cryopreservation (Zhang et al., 2006; Pilbauerova et al., 2021b). Therefore, DPSCs have the potential to be a promising personalized patient-specific stem cells source for regenerative therapy. In this review article, we will discuss the tooth anatomy and dental stem cells with a particular interest on the current advances in adult human DPSCs including their origin, biological characteristics, heterogenicity, differentiation, and immunomodulatory potentials, as well as paracrine effects and pre-clinical and clinical applications.

## Anatomical Structure of the Tooth

**FIGURE 1:**
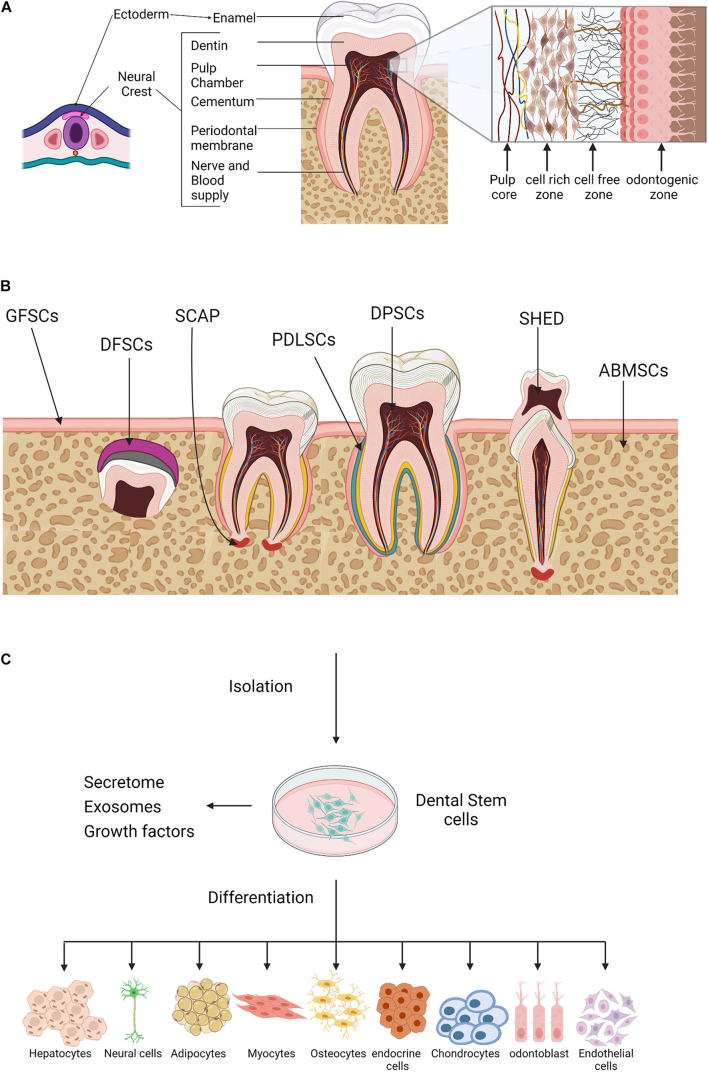
Oral tissue and dental stem cells. **(A)** A schematic outline for the tooth development. Ectoderm cells contribute to the formation of the tooth enamel only, whereas, neural crest cells generate the rest of the tooth tissues. **(B)** Sources of oral tissue and dental stem cells. GMSCs, gingiva-derived mesenchymal stem cells; DFPCs, dental follicle precursor cells; SCAP, stem cells from apical papilla; PDLSCs, periodontal ligament stem cells; DPSCs, dental pulp stem cells; SHED, stem cells from human exfoliated deciduous teeth, ABMSCs, alveolar bone–derived mesenchymal stem cell. **(C)** A schematic illustration of the dental stem cells multilineage differentiation potential. Created with BioRender.com.

Teeth are viable organs made up of well-organized structures with numerous but defined specific shapes (Magnusson, 1968). Odontogenesis or teeth generation undergoes several complex developmental stages that are yet to be fully defined (Smith, 1998; Zheng et al., 2014; Rathee and Jain, 2021). Remarkably, the tooth tissues originate from different cell lineages. The enamel develops from cells derived from the ectoderm of the oral cavity, whereas the cementum, dentin, and pulp tissues are derived from neural crest-mesenchyme cells of ectodermal and mesodermal origins (Figure 1A; Miletich and Sharpe, 2004; Thesleff and Tummers, 2008; Caton and Tucker, 2009; Koussoulakou et al., 2009). The lineage diversities may explain the observed differences in tissue topography and physiological function. The enamel-producing cells and associated metabolites are lost during tooth eruption, whereas pulp cells are longevous and have the capacity to undergo remodeling and regeneration (Simon et al., 2014).

The dental pulp is a highly vascularized connective tissue, consists of four zones, namely (1) the peripheral odontogenic zone, (2) intermediate cell-free zone, (3) cell-rich zone, and (4) the pulp core (Figure 1A, insert). Adjacent to the dentin layer, the peripheral odontogenic zone contains the specialized columnar odontoblast cells that produce dentin (Gotjamanos, 1969; Sunitha et al., 2008; Pang et al., 2016; Ghannam et al., 2021). Besides the extensive vascular and neuronal network, the cell-free zone or basal layer of Weil contains a small number of cells whereas, high density of specialized cells is observed in the underlying zones. The cell-rich zone contains fibroblasts, macrophages, capillaries and proliferating mesenchymal cells that can differentiate into odontoblasts. The pulp core is populated by multiple populations of dental mesenchymal cells, mesenchymal-like stem cells, macrophages and dendritic cells that maintain the dentin-pulp complex functionality and homeostasis (Berkovitz, 1989; Abd-Elmeguid and Yu, 2009; Farges et al., 2015).

## Stem Cells in the Tooth Compartments

Like other organs, tooth compartments harbor a niche of heterogeneous stem/progenitor cell populations of the embryonic stem cells; however, the developmental stage for most dental stem cells has not been established yet and their precise role remains poorly understood (Kaukua et al., 2014; Krivanek et al., 2017). Several studies have indicated that in mild tooth trauma and post-inflammatory recovery, these cells regenerate dentin barrier to protect the pulp from infectious agents and demonstrate an immunomodulatory capacity, either via secreting proinflammatory cytokines or through crosstalk with immune cells (Lesot, 2000; Tomic et al., 2011; Hosoya et al., 2012; Leprince et al., 2012; Li et al., 2014).

The various sources of dental progenitor cells include the DPSCs (Gronthos et al., 2000), stem cells from human exfoliated deciduous teeth (SHED) (Miura et al., 2003), periodontal ligament stem cells (PDLSCs) (Seo et al., 2004), dental follicle stem cells (DFSCs) (Morsczeck et al., 2005), stem cells from apical papilla (SCAP) (Sonoyama et al., 2006, 2008), and gingival stem cells (GING SCs) (Mitrano et al., 2010; Figure 1B). Like bone marrow-derived mesenchymal stem cells (BM-MSCs), dental progenitor/stem cells exhibit self-renewal capacity and multilineage differentiation potential. *In vitro* studies have shown that dental stem cells generate clonogenic cell clusters, possess high proliferation rates and have the potential of multi-lineage differentiation into a wide spectrum of cell types from the three germ layers or, at least in part, express their specific markers under the appropriate culture conditions (Figure 1C). Despite being similar at a coarse level, the transcriptomic and proteomic profiles of oral stem cells reveal several molecular differences including differential expression of surface marker, structural proteins, growth hormones, and metabolites; indicating prospective developmental divergence (Hosmani et al., 2020; Krivanek et al., 2020), and also suggest that dental stem cells might be the optimal choice for tissue self-repair and regeneration.

## Dental Stem Cell Lineage Tracing

The plasticity and multi-potential competency of oral stem cells owe to the fact that the dental pulp contains neuro-mesenchymal components. Genetic lineage tracing studies have identified the perivascular pericytes and glial cells as immediate ancestors of the dental stem cells. Utilizing mouse incisors’ regenerative capacity as a model for recovery from pulp/dentin injury, Feng et al. (2011) identified the pericytes NG^+^ cells as oligodendrocyte progenitors. Post trauma, pericytes NG^+^ cells proliferate rapidly and partially contribute to the generated odontoblasts (Pang et al., 2016). Later, Zhao et al. (2014) reported the role of neurovascular sensory cells in activating periarterial Gli1^+^ cells through sonic hedgehog signaling pathways, which were sufficient to maintain homeostasis and injury repair of the incisor mesenchyme. Notably, lineage tracing studies revealed that Gli1^+^ cells contributed to the entire pericytes NG^+^ cell population, but not vice versa (Zhao et al., 2014). Therefore, periarterial Gli1^+^ cells are believed to be the sole source of odontoblast derivation. These observations were further supported experimentally by combining a clonal color-coding technique with tracing of peripheral glia cells, in addition, quantification analysis revealed that both pericytes and glial cells contribute equally to the dynamics of tooth organogenesis, homeostasis, and growth (Kaukua et al., 2014; Sharpe, 2016; Shi et al., 2020).

## Dental Pulp Stem Cells Isolation Procedures and Culture Conditions

Dental pulp stem cells constitute merely 5% of the pulp cells and they were first isolated and characterized by Gronthos et al. (2000). The quality of the isolated DPSCs primarily impacts their regenerative potential. The culturing method and accurate characterization are pivotal steps for the isolation of high-quality DPSCs. Following extraction of the third molar, further procedures include mechanical extraction of the soft pulp connective tissue, maceration, enzymatic digestion of extracellular matrix proteins (ECM), and cell growth in plastic tissue/cell culture plates. The various isolation and culture procedures used for the human DPSCs have been best reviewed by Rodas-Junco and Villicana (2017).

Here, we also describe the standard procedure used in our clinic and laboratory. Briefly, immediately after extraction, the third molar is thoroughly rinsed with ethanol and sterile distilled water. Using a cylindrical turbine bur, an incision is made between the enamel and the cement at the point of molar fracture. The fragmented tooth is refreshed in PBS in sterile tubes and rushed to the laboratory. Using aseptic techniques, the tooth is transferred to a petri dish and dental pulp tissue is isolated using a sterile nerve-puller file-15 and forceps, chopped into fine fragments and digested by collagenase type I for 60 min at 37°C. Single cell suspension is prepared by first passing cells through an insulin syringe and then passing through cell strainer with 40 μm APD, followed by centrifugation. The cell fraction is washed with sterile PBS, counted and cells are seeded in culture medium. For primary culture establishment, cells are seeded in fibronectin-coated culture dishes. At 60% confluency, DPSCs are passed at a cell density of 80–100 cells/cm^2^. DPSCs expansion medium consists of 60% DMEM-low glucose and 40% chick fibroblast basal medium MCDB-201, supplemented with a myriad of factors such as Insulin-Transferrin-Selenium (ITS), linoleic acid bovine serum albumin (LA-BSA), dexamethasone (dex), ascorbic acid 2-phosphate (Asc-2P), antibiotics (Penicillin/Streptomycin), human Platelet-Derived Growth Factor (hPDGF)-BB, human Epidermal Growth Factor (hEGF), human Leukemia Inhibitory Factor (hLIF), Chemically Defined Lipid Concentrate (CDLC), and β-mercaptoethanol (Atari et al., 2012; Martinez-Sarra et al., 2017; Nunez-Toldra et al., 2017a,b; Al Madhoun et al., 2018; Faruqu et al., 2020).

## Dental Pulp Stem Cells Markers

Dental pulp stem cells are a heterogeneous mixture of cell populations with no distinct cell surface antigens (Kawashima, 2012). DPSCs display characteristics that are much similar to those of MSCs such as the abilities for self-renewal and multilineage differentiation. According to the minimal criteria defined by the International Society of Cellular Therapy (ISCT) for the human MSCs, these cells adhere to plastic, express CD29, CD44, CD49a-f, CD51, CD73 (SH3), CD90, CD105 (SH2), CD106, and CD166, and lack the expression of the hemopoietic surface antigens including CD11b, CD14, CD19, CD34, CD45, CD79a, and human leukocyte antigen-DR isotype (HLA-DR) (Dominici et al., 2006; Sonoyama et al., 2006; Huang et al., 2009; Wu et al., 2015). DPSCs express a wide spectrum of other surface markers also as shown in Table 1. However, notable complexity and divergence in their expression levels have been reported by several groups (Laino et al., 2005; Yamada et al., 2010; Hilkens et al., 2013; Niehage et al., 2016; Alraies et al., 2020) which could be attributed, at least in part, to their heterogenicity. DPSCs can be enriched by using different isolation procedures and cell culture conditions. For example, their surface marker expression may vary depending on the serum concentrations and/or the addition of growth factors to the basal culture media. Martens et al. (2012) have documented expression of the neural markers (nestin, vimentin, synaptophysin, S100, and βIII-tubulin) on undifferentiated DPSCs that were cultured in media containing 10% FBS. Longoni et al. (2020) reported fibrous cartilage tissue conversion (expression of aggrecan, glycosaminoglycan, elevated expression of collagen type I, and limited expression of collagen type II) of DPSCs using chondro-inductive growth factors such as insulin-like growth factor (IGF)-1, transforming growth factor (TGF)-β3, and bone morphogenetic protein (BMP)-2, -6, -7. Notably, Zhang et al. (2008) have reported adipogenic, myogenic, and odontogenic plasticity of the DPSCs using respective lineage-specific pre-inductions media *in vitro*.

**TABLE 1:** Surface markers and pre-lineages expression profile of dental pulp stem cells (DPSCs).

Marker	Protein name	Expression strength	References
**MSCs surface markers**			
Stro-1	Stro-1 antigen remains unidentified	+++	Shi et al., 2005; Jo et al., 2007; Liu et al., 2009; Atari et al., 2011; Huang et al., 2014
CD9 (TSPAN29)	Cell growth-inhibiting gene 2 protein	++	Kawanabe et al., 2012; Niehage et al., 2016
CD13 (ANPEP)	Alanyl aminopeptidase	++	Huang et al., 2009; Atari et al., 2011; Kawanabe et al., 2012
CD29 (ITGB1)	Integrin beta 1	++	Huang et al., 2009; Kawanabe et al., 2012; Niehage et al., 2016; Mendi et al., 2017
CD39 (Entpd1)	Lymphoid cell activation antigen	+	Niehage et al., 2016
CD44	CD44 antigen	+++	Shi et al., 2005; Huang et al., 2009; Kawanabe et al., 2012; Niehage et al., 2016; Mendi et al., 2017
CD49a, b, c, d, e (ITGA1,2,3,4,5)	Integrin alpha-1,2,3,4,5	++	Ferro et al., 2012a; Kawanabe et al., 2012
CD51 (ITGAV)	Integrin alpha V	+	Kawanabe et al., 2012
CD73 (NT5E)	5’-nucleotidase	+++	Huang et al., 2009; Kawanabe et al., 2012; Ponnaiyan et al., 2012; Niehage et al., 2016; Mendi et al., 2017
CD90 (Thy-1)	Thy-1 membrane glycoprotein	+++	Huang et al., 2009; Kawanabe et al., 2012; Ponnaiyan et al., 2012; Niehage et al., 2016; Mendi et al., 2017
CD105 (END)	Endoglin	++	Huang et al., 2009; Ferro et al., 2012a; Kawanabe et al., 2012; Ponnaiyan et al., 2012; Niehage et al., 2016
Cd117 (c-Kit)	Mast/stem cell growth factor receptor Kit	+	Yang et al., 2009; Ishkitiev et al., 2012; Kawashima, 2012
CD146 (MUC18)	Melanoma Cell Adhesion Molecule	+++	Shi et al., 2005; Huang et al., 2009, 2014; Niehage et al., 2016
CD151 (TSPAN24)	Platelet-endothelial tetraspan antigen 3	+++	Niehage et al., 2016
CD166 (ALCAM)	Activated Leukocyte Cell Adhesion Molecule	+++	Kawanabe et al., 2012; Ponnaiyan et al., 2012; Niehage et al., 2016
**Other surface markers**			
CD59	CD59 glycoprotein	+	Huang et al., 2009; Ferro et al., 2012a
ITA10 (ITGA10)	Integrin alpha 10	++	Liu J. et al., 2011; Niehage et al., 2016
ITA8 (ITGA8)	Integrin alpha 8	+	Liu J. et al., 2011; Niehage et al., 2016
CD325 (CDH2)	Cadherin-2	++	Niehage et al., 2016
MFGM	Lactadherin	+	Niehage et al., 2016
**Embryonic stem cells markers**			
OCT4 (POU5F1)	POU domain, class 5, transcription factor 1	+++	Kerkis et al., 2006; Atari et al., 2011; Liu L. et al., 2011; Ishkitiev et al., 2012; Kawanabe et al., 2012; Ponnaiyan et al., 2012; Huang et al., 2014; Wu et al., 2015; Ballini et al., 2019
Nanog	Homeobox protein NANOG	+++	Kerkis et al., 2006; Atari et al., 2011; Liu L. et al., 2011; Ishkitiev et al., 2012; Kawanabe et al., 2012; Ponnaiyan et al., 2012; Huang et al., 2014; Ballini et al., 2019
KLF4	Krueppel-like factor 4	+	Ballini et al., 2019
c-Myc	MYC proto-oncogene, BHLH transcription facto	+	Liu L. et al., 2011; Ballini et al., 2019
Sox2	Transcription factor SOX-2	++	Govindasamy et al., 2010; Atari et al., 2011; Kawanabe et al., 2012; Ballini et al., 2019
Sox1	Transcription factor SOX-1	++	Atari et al., 2012; Ferro et al., 2012a; Tan and Dai, 2017
CD9	Tetraspanin receptor	+++	Lindroos et al., 2008; Ponnaiyan et al., 2012
CD13	Aminopeptidase N	++	Ponnaiyan et al., 2012; Akpinar et al., 2014
SSEA4	Stage specific embryonic antigen 4	++	Kerkis et al., 2006; Atari et al., 2011; Ferro et al., 2012a; Kawanabe et al., 2012; Ponnaiyan et al., 2012
SSEA3	Stage specific embryonic antigen 3	+	Kerkis et al., 2006; Ferro et al., 2012a; Kawanabe et al., 2012
SSEA1	Stage specific embryonic antigen 1	+	Kawanabe et al., 2012
REX1 (ZFP42)	Zinc finger protein 42 homolog, Zfp-42	++	Kawanabe et al., 2012
TERT	Telomerase reverse transcriptase	+	Huang et al., 2009
TRA-1-60	TRA-1-60 antigens	+	Kerkis et al., 2006; Atari et al., 2012
TRA-1-81	TRA-1-81 antigens	+	Kerkis et al., 2006
MixL1	MIX1 homeobox-like protein 1	+	Atari et al., 2012
GATA4	GATA binding protein 4	+	Atari et al., 2012
GATA6	GATA binding protein 6	+	Atari et al., 2012
**Immune markers**			
HLA-ABC	MHC class I antigen	+	Ponnaiyan et al., 2012
**Osteo/odontogenic markers**			
ALP	Alkaline phosphatase	+++	Shi et al., 2005; Huang et al., 2009; Yildirim et al., 2016; Ching et al., 2017
BSP	Bone Sialoprotein 2	+	Sonoyama et al., 2008; Huang et al., 2009
OCN	Osteocalcin	+++	Shi et al., 2005; Sonoyama et al., 2008; Huang et al., 2009; Ching et al., 2017
OPN	Osteopontin	++	Shi et al., 2005
MEPE	Matrix extracellular phosphoglycoprotein	+++	Sonoyama et al., 2008; Huang et al., 2009; Ching et al., 2017
Runx2 (OSF-2)	Osteoblast-specific transcription factor 2	+	Huang et al., 2009; Yildirim et al., 2016
Scleraxis	Tendon specific transcription factor	+	Shi et al., 2005; Huang et al., 2009
**Angiogenic markers**			
Endostatin (COL18A1)	Collagen Type XVIII Alpha 1 Chain	++	Huang et al., 2009; Bronckaers et al., 2013; Hilkens et al., 2014
VEGF	Vascular endothelial growth factor	+	Tran-Hung et al., 2008; Bronckaers et al., 2013; Hilkens et al., 2014; Yu et al., 2016
ANGPT1	Angiopoietin-1	+	Bronckaers et al., 2013; Hilkens et al., 2014; Yu et al., 2016
IFGBP3	Insulin-like growth factor binding protein 3	+	Bronckaers et al., 2013; Hilkens et al., 2014; Yu et al., 2016
PTX3	Pentraxin-3	+	Bronckaers et al., 2013; Hilkens et al., 2014; Bakopoulou et al., 2015
PAI-1	Plasminogen activator inhibitor 1	+++	Bronckaers et al., 2013; Hilkens et al., 2014; Yu et al., 2016
TIMP1	Tissue inhibitor of matrix metalloproteinase 1	+++	Bronckaers et al., 2013; Hilkens et al., 2014; Bakopoulou et al., 2015; Yu et al., 2016
PDGF	Platelet-derived growth factor	+	Tran-Hung et al., 2008
**Growth factors receptors**			
TGFβRII	Transforming growth factor beta receptor 2	+	Huang et al., 2009; Karaoz et al., 2011
FGFR3	Fibroblast growth factor receptor 3	+++	Huang et al., 2009
EGFR	Epidermal growth factor receptor	+	Niehage et al., 2016
Flt-1 (VEGFR1)	Vascular endothelial growth factor receptor 1	+	Huang et al., 2009
FGFR1	Fibroblast growth factor receptor 1	+++	Huang et al., 2009
CD271 (NGFR)	Low-affinity nerve growth factor receptor	+	Nakashima et al., 2009; Mikami et al., 2011
**Neuronal markers**			
Nes	Nestin	+	Huang et al., 2009; Martens et al., 2014; Yildirim et al., 2016
bFGF (FGF2)	Fibroblast growth factor 2	++	Tran-Hung et al., 2008; Huang et al., 2009
BDNF	Brain-derived neurotrophic factor	++	Atari et al., 2012; de Almeida et al., 2014
NFM (NEF3)	Neurofilament medium polypeptide	+	Huang et al., 2009; Martens et al., 2014; Yu et al., 2016
GDNF	Glial cell derived neurotrophic factor	+	Huang et al., 2009; Martens et al., 2014; Yu et al., 2016
NGF	Nerve growth factor	+	Huang et al., 2009; Martens et al., 2014; Yu et al., 2016
NCAM2	Neural Cell Adhesion Molecule 2	+	Martens et al., 2014; Niehage et al., 2016; Yu et al., 2016
Slug	Neural crest transcription factor slug	++	Huang et al., 2009
TUBB3	Tubulin beta 3 Class III	++	Huang et al., 2009

Antibody-based methods, proteomics and RNA transcriptomics are the main procedures used for DPSCs immunophenotyping. Besides the MSCs markers, DPSCs possess the embryonic stem cell-specific markers (Table 1). In addition, DPSCs express a variety of antigens associated with cell adhesion, growth factors, transcription regulation and multiple lineage-specific markers related to perivascular tissue, endothelium, immunogenic, neuronal and osteo/odontogenic tissues (Table 1). It is also noteworthy to mention that DPSCs express Major Histocompatibility Complex (MHC) class I antigens, but they do not express the immune co-stimulating molecules such as MHC class II antigen HLA-DR, CD40, CD80, and CD86 (Wada et al., 2009; Bhandi et al., 2021; Pilbauerova et al., 2021a).

## Heterogeneity of Dental Pulp Stem Cells

The heterogeneity of the DPSCs subpopulations isolated from different donors is mainly influenced by donor health, age, genetic, and environmental factors (Kellner et al., 2014; Wu et al., 2015; Alraies et al., 2017; Kobayashi et al., 2020; Longoni et al., 2020). Alternatively, the intra-population heterogeneity refers to the DPSCs subpopulations found within the preparation from a single individual. The evidence demonstrating that DPSCs populations are functionally heterogeneous comes largely from their surface antigen profile or expression patterns of a variety of markers that are associated with progenitors of different lineages. DPSCs show surface expression of STRO-1, CD13, CD29, CD44, CD73, CD90, CD105, CD146, and CD166 – a profile which is reminiscent of BM-MSCs (Table 1), while DPSCs lack the expression of hematopoietic (CD34 and CD45) and monocytic (CD14) markers (Yamada et al., 2010). Additionally, DPSCs express various pluripotency markers, such as Oct-3/4, Nanog, and Sox-2, i.e., the stemness-related markers observed in embryonic stem cells (Table 1) which explains, at least in part, their self-renewal potential (Atari et al., 2011, 2012; Ferro et al., 2012b; Faruqu et al., 2020). As neuronal crest-derived cells, DPSCs express several neural stem cell markers, including nestin, neuronal nuclei antigen, vimentin, synaptophysin, musashi-1, Galactosyl-ceramidase, S100 calcium binding protein B, neurofilament heavy (NFH) chain, class III β-tubulin, and neurofilaments (Table 1). Thus, DPSCs are comprised of the progenitor cells that are marked by diverse characteristics, such as clonal heterogeneity, multi-lineage differentiation, self-renewal capacity, and phenotypic complexity.

Notably, specific conditions and media components used may act as a source of potential phenotypic and functional changes in the freshly extracted DPSCs. The isolation procedures may also influence their heterogeneity. Whereas, DPSCs isolation by enzymatic digestion provides a large number of cells at low passage rate, the tissue explants enable the isolation of a more homogeneous cell population (Bronckaers et al., 2013; Raoof et al., 2014). Furthermore, DPSCs heterogeneity is impacted by the culture media components, serum concentration, and growth factors supplements, all of which have been well-reviewed by Rodas-Junco and Villicana (2017). Moreover, long-term and large-scale expansion in culture may also impact the heterogeneity, survival, and differentiation potential of DPSCs. The selection and expansion of different DPSCs subpopulations driven by specific culture conditions, media supplementation and 2D/3D culture systems may collectively alter the cellular profile, homeostasis, plasticity, and regenerative potential, as well as immunomodulatory properties of DPSCs (further elaborated below).

## Dental Pulp Stem Cells Crosstalk With Microenvironment in Homeostasis

Dental pulp stem cells are located within a heterogenic niche. Homeostatic regulation of the DPSCs niche, DPSCs’ proliferation and differentiation implicate a complex network of bioactive molecules, growth factors, ECM, and key signaling pathways (Scheller et al., 2008; Mitsiadis et al., 2011; Tsutsui, 2020; Deng et al., 2021). Nevertheless, the signals that regulate DPSC fate are not only the biochemical cues, but also the biophysical cues (mechanical signals) that play a crucial role in influencing DPSC fate since orthodontic mechanical tension or stresses are exerted to teeth and transmitted into the dental pulp tissue by jaw movement during the process of normal mastication (Tatullo et al., 2016). Thus, DPSCs are mechanosensitive cells by default with the capacity to recognize mechanical signals and transform these stimuli into various cellular responses to sustain niche homeostasis (Han et al., 2008, 2010; Hata et al., 2013). Importantly, Marrelli et al. (2018) have presented an excellent review of the mechanobiology and mechanoresponsiveness of DPSCs, deciphering how the mechanical stimuli might regulate behavior, fate, and homeostasis of DPSCs. These studies may enhance our understanding and improve approaches to the DPSC-based tissue engineering applications.

## Dental Pulp Stem Cells Potential Role in Tissue Repair and Flourishment

Dental pulp stem cells could be valuable source for cell therapy and advancing the current regenerative medicine strategies. DPSCs plasticity to surrounding environment has made them a notable source for disease treatment, though the full understanding of DPSCs tissue repair mechanisms is still in their preliminary stages. In this section, we will review the current advances in DPSCs *in vitro* differentiation potentials and the capability to secrete growth factors that may contribute to their role in tissue repair.

### Differentiation Potential of Dental Pulp Stem Cells

Due to their potential to differentiate into several cell lineages (Figure 1C), DPSCs have received extensive attention in the field of regenerative medicine and tissue engineering. DPSCs have the potential to differentiate into endodermal (respiratory and gastrointestinal tracts, liver, pancreas, thyroid, prostate, and bladder lineages), mesodermal (adipogenic, osteogenic, and chondrogenic lineages) and ectodermal (skin and neural lineages) (Yamada et al., 2019). In addition, DPSCs were shown to differentiate into myocytes, cardiomyocytes, hepatocyte-like cells, melanocytes, and active neurons (Figure 1C; Stevens et al., 2008; Patil et al., 2014). As a thumb rule, a substantial improvement in the efficacy DPSCs differentiation was observed using defined conditioned media.

Several recent reviews have documented the current knowledge and understanding of DPSCs’ differentiation into vital lineages including their angiogenic and neurogenic potential (Ratajczak et al., 2016; Mortada et al., 2018; Mattei et al., 2021), odontogenic and chondrogenic potential (Nuti et al., 2016; Ching et al., 2017; Mortada and Mortada, 2018), and periodontal and dental tissue regeneration (Hu et al., 2018; Zhai et al., 2019); Therefore, we will focus in this section in summarizing the current knowledge regarding DPSCs hepatogenic and pancreatic β-cells differentiation capacities.

#### Differentiation of Dental Pulp Stem Cells Into Hepatocytes

Implementing defined, serum-free, and stepwise differentiation protocols that mimic the developmental stages of hepatocytes during embryogenesis were found to be sufficient for inducing hepatogenesis (summarized in Table 2). Using this approach, Ishkitiev et al. (2010) were the pioneers to demonstrate the hepatogenic differentiation potential of DPSCs. Initially, they developed the Ishkitiev et al. (2010) developed the two-stage conditioned media that contained low percentage of fetal bovine serum (FBS) but was enriched with essential hepatogenic inducers (see Table 2). Later, they used a serum-free conditioned medium to generate the hepatocyte-like cells from a CD117^+^ DPSCs subpopulation (Ishkitiev et al., 2012). The latter hepatogenesis protocol utilized three developmental stages, i.e., cell specification, differentiation, and maturation to generate cells with phenotypical, and functional characteristics similar to hepatocytes. This approach was further improved and implemented in later studies (Table 2; Ferro et al., 2012a; Kumar et al., 2017). Recently, we implemented a similar approach to differentiate a pluripotent-like subpopulation of DPSCs into hepatocyte-like cells with detailed characterization of each differentiation stage and associated markers (Gil-Recio et al., 2020).

**TABLE 2:** Protocols and composition of media used by different studies to generate hepatocytes and insulin producing pancreatic β-cells from DPSCs.

Differentiation	Inducers	Confirmation markers and tests	Protocol timeline (days)	References
Hepatogenesis	• Specification: 2%FBS, HGF • Maturation: ITS-X, dex, and OSM	α-fetoprotein, albumin, hepatic HNF-4, IGF-1, glycogen storage, carbamoyl phosphate synthetase, glucagon, and urea secretion	22–28	Ishkitiev et al., 2010
	• Specification: ITS-X and embryo-trophic factor • Differentiation: ITS-X, embryo-trophic factor, and HGF • Maturation: ITS-X, embryo-trophic factor, HGF, dex, and OSM	α-fetoprotein, albumin, hepatic HNF-4, IGF-1 gene expression. Glycogen storage. Carbamoyl phosphate synthetase, glucagon, and urea secretion	22–28	Ishkitiev et al., 2012
	1% FCS; HGF, oncostatin, nicotinamide, LDL, FGF-4, insulin, glucose, and linoleic acid	Mdr-1, cyp-2e1, Erythropoietin, cytokeratin 8, cytokeratin 18, and cytokeratin 19 gene expression. Albumin secretion	40	Ferro et al., 2012
	• Specification: HGF, EGF, and dex • Maturation: ITS, OSM, and dex	Albumin, TAT, and α-fetoprotein secretion	28	Kumar et al., 2017
	• Definitive endoderm generation: KOSR and Act A • Specification: KOSR, FGF-4, and HGF • Differentiation: FGF-4, HGF, OSM, and dex	AAT, G6P, HNF6, and cytokeratin 18 gene expression. CYP3A4 activity and AST activity. Albumin secretion and Glucagon storage	22	Gil-Recio et al., 2020
Pancreatic β-cell like	• Induction: ITS, Act A, sodium butyrate, and β-ME • Differentiation: ITS, taurine, GLP-1, nicotinamide, and NEAAs	C-peptide, Pdx-1, Pax4, Pax6, Ngn3, and Isl-1 gene expression. Insulin and c-peptide secretion	10	Govindasamy et al., 2011; Sawangmake et al., 2014; Kanafi et al., 2013; Matei et al., 2017; Yagi Mendoza et al., 2018
	• Pre-induction: FBS, RA, and β-ME • Induction: nicotinamide, RA, and β-ME. Alternation between L-DMEM and H-DMEM media, each supplemented with FBS, NNAA, zinc sulfate, and selenium	PDX-1, insulin and GLUT-2 gene expression. Dithizone staining. Insulin secretion	21	Carnevale et al., 2013
	• Induction: HGF and αFGF • Differentiation: HGF, αFGF, EGF, and β-ME. Maturation: HGF, nicotinamide, and embryo-trophic factor	Generation of pancreatic -a, -b, -d, and pancreatic polypeptide-producing cells. Expression of Pdx-1Hhex, Mnx1, Neurog3, Pax4, Pax6, Nkx6-1, Ins, GLG, PPY, SST, Glut-2 and AMY2A, and insulin	17	Ishkitiev et al., 2013
	Matrigel (3D biomaterial matrix) • Stage 1: Act A, Noggin, LiCl, polyvinyl chloride, and β-ME • Stage 2: retinoic acid, A83-01, LDE225, A83-01, and polyvinyl chloride • Stage 3: polyvinyl chloride, ITS, SB, nicotinamide, and NNAAs	Gene expression for FOXA1, Sox17, glucagon, Pax4, Insulin, Nkx6-1, Neurog3, NeuroD1, Pdx1, and CXCR4. Immunohistochemistry for Nkx6-1, Pdx-1, CXCR4, Sox17, and insulin	Not mentioned	Xu et al., 2019

#### Differentiation of Dental Pulp Stem Cells Into Pancreatic Insulin-Producing Cells

There are several reports (summarized in Table 2) describing the differentiation of DPSCs into glucose-responsive pancreatic insulin-producing β-cells (IPCs). Using a three-step differentiation procedure, IPCs induction and functionality were confirmed by insulin secretion and C-peptide expression in a glucose-dependent manner (Table 2; Govindasamy et al., 2011; Carnevale et al., 2013; Sawangmake et al., 2014). Interestingly, another study demonstrated that DPSCs-derived IPCs were physiologically functional and, as expected, reversed hyperglycemia to the normal level in streptozotocin (STZ)-induced diabetic mice (Kanafi et al., 2013). Furthermore, Matei et al. (2017) observed that hydrogen sulfide exposure increases insulin and C-peptide secretions, protects against glucotoxicity, and enhances the expression insulin and PI3K/AKT pathway. As a proof of principle, Ishkitiev et al. (2013) confirmed that CD117^+^ DPSCs subpopulation generated a heterogeneous population of cells that expressed pancreatic-specific endocrine and exocrine markers (Table 2). Notably, comparative studies between 2D and 3D culture systems revealed that IPCs in 3D models mimic *in vivo* cell growth and possess phenotypical structures like native pancreatic islets (Yagi Mendoza et al., 2018; Xu et al., 2019). Importantly, Xu et al. (2020) documented that coating IPCs with Matrigel, a basement membrane matrix, improves cell survival after orthotopic injection into the pancreatic parenchyma of Sprague Dawley (SD) rats (Table 3).

**TABLE 3:** Immunomodulatory profile of DPSCs reported in different studies.

Cell Types	Stimuli	Methods	Outcomes	References
T-lymphocytes were isolated from peripheral blood of healthy donors	PHA	Co-culture	Suppression of activated T-cell	Pierdomenico et al., 2005
CD4+-Tbet+(Th1) and CD4+-Gata3+(Th2)	PHA	Co-culture	Suppression of IFN-γ and reduction of IL-4	Ozdemir et al., 2016
CD4+-Stat3+ (Th17) and CD4+-CD25+-FoxP3+(Treg)			Stimulated, IL-17 increases	
CD4+ CD25+ FoxP3+ T cells			No effect, induction of TGFb1 andIL-10	
PBMNC	Con A or MLR	Co-culture	Proliferation inhibition and cell cycle arrest at G0	Wada et al., 2009
	IFN-γ pre-treated DPSCs conditioned medium	Trans-well, conditioned media (CM) pretreatment with INF-γ		
CD3 T cells	PHA or MLR	Co-culture Transwell	• Proliferation inhibition and apoptosis induction • Inhibition of IL-2, IL-6, IL-12, IFN-γ, and TNF-α • Induction of anti-inflammatory CD4 CD25 Foxp3, and CXCL10	Demircan et al., 2011
CD8+ T lymphocytes, B lymphocytes	PHA or MLR. Antibodies against CD3 and CD28	Co-culture Transwell	• Allogeneic proliferation inhibition • Inhibition of PBMCs response to stimuli • Abrogation of IgM and IgG production by allogeneic B cells	Kwack et al., 2017
THP-1 cells differentiated into macrophages	LPS, nigericin	Co-culture Transwell	• Abrogation of LPS-stimulated secretion of TNF-α but not IL1B • Increase in IDO protein expression • Decrease in p-NFκB-p65 (ser486) expression level	Lee et al., 2016

## Paracrine Activity of Dental Pulp Stem Cells: Secretome and Exosomes

Currently, accumulating evidence indicates that a great deal of therapeutic benefit of primary DPSCs exists in their paracrine activity, that is, the ability to modulate their microenvironment through the release of bioactive molecules. These factors can be released directly into the surrounding microenvironment known as secretome or they can be embedded within the membrane-bound extracellular nanovesicles (∼30–150 nm in diameter), known as exosome (Thery et al., 2002). These factors include cytokines, chemokines, growth factors, angiogenic mediators, hormones, and regulatory nucleic acid molecules (Wei et al., 2020). In general, secretome and exosomes participate in the processes of tissue replenishment, cellular homeostasis, anti-inflammation, immunomodulation, and other functions (Tang et al., 2021).

In relation to tumor tropism, Altanerova et al. (2016) engineered DPSCs to express the fused yeast suicide gene cytosine deaminase::uracil phosphoribosyl transferase (yCD::UPRT), a gene that converts the nontoxic 5-fluorocytosine (5-FC) into the toxic 5-fluoro-20-deoxyuridine-50-monophosphate (5-FdUMP) (Graepler et al., 2005). Exosomes released from these engineered DPSCs were sufficient to integrate human tumor cells transplanted into the brain of rat models and were able to induce apoptosis in the presence of pro-drug 5-FC (Altanerova et al., 2016).

Secretome of DPSCs contains neurotrophic factors (NDNF, NT-3, NGF, and GDNF) and TGF-β, possessing the therapeutic potential for neurodegenerative diseases (de Almeida et al., 2011). In Alzheimer’s disease (AD) cell model, DPSCs’ secretome reduced the amyloid beta (Aβ) peptide-mediated cell cytotoxicity and apoptosis by stimulating the endogenous survival factor Bcl-2 and decreasing the apoptotic regulator Bax (Ahmed Nel et al., 2016). DPSCs secretome promoted proliferation, migration, and survival in Schwann peripheral glial cells (Yamamoto et al., 2016) and SH-SY5Y neuroblastoma cells (Gervois et al., 2017). Furthermore, conditioned media from DPSCs improved neuromuscular junction innervation and motor neuron survival in a mouse model of amyotrophic lateral sclerosis (ALS) (Wang et al., 2019), and promoted the survival of retinal ganglion cells in a rat model for optic nerve injury (Mead et al., 2014).

Dental pulp stem cells secrete angiogenic growth factors including VEGF, bFGF, and PDGF which are sufficient to mediate the formation of a network of tubular structures of endothelial cells, an indicator of angiogenic stimulation (Zhou H. et al., 2020). Furthermore, conditioned media from cultured DPSCs promoted wound healing, angiogenesis and soft-tissue regeneration in a mouse model of excisional wound healing (Yang et al., 2013).

Taken together, DPSCs secretome and exosome are rich in trophic factors that can mediate tissue regeneration and proliferation, which could be highly useful for prospective cell-free regenerative medicine and advantageous over interventions involving cell transplantation (Vizoso et al., 2017). Nevertheless, a proper characterization of the DPSCs’ secretome and exosome is required since they are significantly influenced by culture conditions (Chin et al., 2021), hypoxia (Aranha et al., 2010), insult (Mattei et al., 2021), DPSCs passage (Faruqu et al., 2020), subpopulation (Nakashima et al., 2009), and stage of differentiation (Huang et al., 2016).

## Immunomodulatory Properties of Dental Pulp Stem Cells

The crosstalk between DPSCs and immune cell subsets impacts the functioning of both the innate and adaptive immune systems, implying that DPSCs have immunomodulatory properties – an exciting field that needs to be further investigated [best reviewed in Li et al. (2014) and Andrukhov et al. (2019)]. Immunomodulatory phenotype of DPSCs is primarily attributed to *in vitro* cell culture approaches and conditions, such as enzymatic stimuli, soluble factors secretions, and cell-to-cell contacts. As though, all of these attributes may not precisely mimic the complexity of the *in vivo* microenvironment, nevertheless, the data generated are valid, at least in part, with respect to the studied immune cell subsets.

Co-culture cell models have revealed that DPSCs mediate G0/G1 cell cycle arrest of the chemically-activated T cells (Table 3; Pierdomenico et al., 2005); while, other studies also show induction of differential T-cell subset responses. Co-cultures of DPSCs with CD3^+^, CD4^+^, or CD8^+^ T cells mediated differential proliferation arrest, apoptosis and/or induction of regulatory T cells (Treg) (Table 3; Demircan et al., 2011; Zhao et al., 2012; Ozdemir et al., 2016; Kwack et al., 2017). Interestingly, proliferation inhibition of the peripheral blood mononuclear cells (PBMCs) was observed in cultures with conditioned medium from DPSCs pre-treated with interferon (INF)-γ (Wada et al., 2009). Taken together, T lymphocyte activation and INF-γ production is a prerequisite for the induction of immunomodulatory DPSCs and secretion of IL-10/TGF-β1, expression of soluble factors inducing Treg formation and lymphocytes proliferation arrest (Ding et al., 2015; Kwack et al., 2017). Furthermore, co-culture of the DPSCs isolated from symptomatic irreversible pulpitis with macrophages suppresses the LPS-stimulated secretion of TNF-α, via TNF-α/IDO (indoleamine 2,3-dioxygenase) axis mechanism (Lee et al., 2016). In a rat model of diabetic neuropathy, DPSCs transplantation led to the anti-inflammatory M2-type macrophage polarization and ameliorated diabetic polyneuropathy (Omi et al., 2016). In addition, DPSCs were reported to express the complement cascade receptors C3aR and C5aR, and the treatment with C3a or C5a augmented DPSCs’ proliferation and mobilization (Chmilewsky et al., 2014; Rufas et al., 2016).

## Pre-Clinical and Clinical Applications of Human Dental Pulp Stem Cells

Dental pulp stem cells have remarkable potential as alternative sources to multipotent MSCs and due mainly to their immunomodulatory properties as discussed earlier, DPSCs constitute a highly valuable source for cell therapy of a variety of inflammatory diseases and other disorders. Briefly, in regard to basic or pre-clinical studies in experimental animal models, the human DPSCs-based therapies, implicating cells or secretome/conditioned media, have been successfully used in several disease conditions, which has been extensively reviewed by Anitua et al. (2018) including diabetes (Govindasamy et al., 2011; Datta et al., 2017), neuropathy (Makino et al., 2019), hepatic diseases (Cho et al., 2015; Kim et al., 2016), oculopathies (Syed-Picard et al., 2015; Kushnerev et al., 2016; Mead et al., 2016), spinal cord injury (Sakai et al., 2012; Yang et al., 2017), peripheral nerve injury (Sasaki et al., 2011; Sanen et al., 2017), AD (Nakashima et al., 2009; Mita et al., 2015; Wang et al., 2017), cerebral ischemia (Leong et al., 2012; Song et al., 2017), muscular dystrophy (Kerkis et al., 2008; Pisciotta et al., 2015; Martinez-Sarra et al., 2017), myocardial infarction (Gandia et al., 2008), Parkinson’s disease (Nesti et al., 2011; Gnanasegaran et al., 2017a,b), lung injury (Wakayama et al., 2015), and stroke (Yang et al., 2009; Leong et al., 2012; Song et al., 2015). It is noteworthy that not many clinical trials have so far published their results. Table 4 summarizes the clinical investigations that have ingeniously tested the regenerative potentials and multifaceted benefits of human DPSCs in various trials.

**TABLE 4:** Pre-clinical/clinical studies involving use of human DPSCs.

Study type	Description/approach	Methods & outcome	References
Pre-clinical comparative study using a canine bone defect model	Comparative study aimed to investigate the cell-based bone engineering efficacy and determined the association between the osseointegration of dental implants and tissue-engineered bone by using DPSC, BM-MSC, and periosteal cells (PC)	All premolars and the first molar were extracted from 3 dogs. In each animal, 6 bone defects, 3 on either side, were created after 4 weeks. Different materials were implanted in the defects and allowed to heal. Dental implants were placed in the defects after 8 weeks. After 8 more weeks, bone regeneration was assessed by histology and histomorphometry. It was concluded that DPSC had the highest osteogenic potential compared to BMSC and PC, proving them as a valuable cell source for tissue-engineered bone around dental implants	Ito et al., 2011; Martens et al., 2014
Clinical study investigating bone regeneration effects	Bone regeneration capacity, comparative study between DPSCs, DTSC and BM-MSCs, on hydroxyapatite-coated osseointegrated dental implants, by using tissue engineering technology	*In vitro*, human DPSCs and DTSCs expressed osteogenic marker genes including alkaline phosphatase, Runx2, and osteocalcin. *In vivo*, the prepared bone defect model was implanted with graft materials. After 8 weeks, the dental implants were installed and after 16 weeks, sections were assessed histologically and histometrically, confirming the presence of well-formed mature bone and neovascularization. Stem cells with platelet-rich plasma (PRP) can generate bone which might be useful for osseointegrated hydroxyapatite-coated dental implants with improved levels of bone-implant contact	Yamada et al., 2010
Pilot clinical study to assess the safety, efficacy, potential, and feasibility of autologous transplantation of mobilized DPSCs in pulpectomized teeth	Five patients with irreversible pulpitis were enrolled and followed up for up to 24 weeks after mobilized DPSC transplantation	Mobilized DPSCs were procured from discarded teeth and then expanded. The quality of mobilized DPSCs at 9 or 10 passages was assessed by karyotyping. Mobilized DPSCs were transplanted with GCSF in atelocollagen into pulpectomized teeth. No adverse events or toxicity was observed. The cone beam computed tomography confirmed functional dentin formation in 3/5 patients. This study concluded that human mobilized DPSCs were safe and efficacious for total pulp regeneration in endodontics in humans	Nakashima et al., 2017; Karaoz et al., 2011; Mikami et al., 2011
A clinical study using a biocomplex constructed from DPSCs and a collagen sponge scaffold for oro-maxillo-facial (OMF) bone tissue repair in patients requiring extraction of their third molars	This study involved the patients with bilateral bone resorption of the alveolar ridge distal to the 2nd molar, secondary to impaction of the 3rd molar on the cortical alveolar lamina. Since this clinical condition does not permit spontaneous bone repair after extraction of the third molar, it eventually leads to loss of the adjacent 2nd molar as well	DPSCs were isolated from the extracted maxillary 3rd molars and the cells were seeded onto a collagen sponge scaffold. DPSC/collagen sponge biocomplex was used to fill in the injury site. After 3 months of the autologous DPSCs grafting, alveolar bone showed optimal vertical repair and complete restoration of periodontal tissue. Histology revealed the complete bone regeneration, with optimal results after 1 year of autologous DPSCs grafting, indicating that these cells could be used for the repair and regeneration of tissues and organs	D’Aquino et al., 2009; de Almeida et al., 2014
Clinical study evaluating the biological and clinical implications at 3 years following the DPSC-based transplants in human mandibles	This study investigated the stability and quality of the regenerated bone and vascularization after 3 years of the grafting intervention	The authors used conventional procedures, in-line holotomography, and advanced phase-imaging method using synchrotron radiation for increased sensitivity toward low-absorbing structures. It was observed that the regenerated tissue from the graft sites comprised of a fully compact bone with a higher matrix density than control human alveolar spongy bone from the same patient. Although the regenerated bone was not of the proper type found in the mandible, it had a positive clinical impact in terms of increased implant stability as well as improved resistance to physical, chemical, mechanical and pharmacological agents	Giuliani et al., 2013
A 1-year follow-up case series that explored the potential clinical benefits of the DPSCs’ in the regenerative treatment of deep intra-bony defects	In 11 chronic periodontitis patients, a total of 11 isolated intra-bony defects were accessed with a minimally invasive flap and were filled with autologous DPSCs, seeded on a collagen sponge	At 1 year of autologous DPSCs implants, an average clinical attachment level gain of 4.7 ± 1.5 mm, associated with a residual mean probing depth (PD) of 3.2 ± 0.9 mm and remarkable stability of the gingival margin was attained. In 63.6% of the experimental sites, complete pocket closure (PD < 3 mm) was achieved. The clinical outcomes, as supported by the radiographic analysis, showed a bone fill of 3.6 ± 1.9 mm	Aimetti et al., 2018
A single center, two arm ratio 1:1, triple blinded, randomized, placebo-controlled, parallel group, clinical trial (phase I/II study)	The study enrolls 20 serious COVID-19 cases (18–65 years), diagnosed with severe pneumonia: nucleic acid test SARS-CoV-2 positive; respiratory distress (respiratory rate > 30 times per min); hypoxia (resting oxygen saturation < 93% or arterial partial pressure of oxygen/oxygen concentration < 300 mmHg); and typical lung lesions confirmed in chest X-ray image	Both the experimental and control groups receive necessary routine treatment for COVID-19. The experimental group receives the human DPSCs suspension intravenously (3.0 × 10^7^ cells in 30 mL saline solution) on days 1, 4, and 7; while the control group receives an equal amount of saline only (placebo) in parallel. Clinical and laboratory observations (blood tests, liver and kidney functions, inflammatory markers, and immunological tests) to be performed during a period of 28 days for each individual. The primary outcome is time to clinical improvement, i.e., the time (days) it takes to downgrade two levels from the following six ordered grades: Grade 1 – discharge and Grade 6 – death	Ye et al., 2020
Interventional clinical trial of DPSCs (Single group assignment model)	Recruits’ adults/older adults (18–75 years). The experimental arm receives the MSC infusions at days 1, 3, and 7. The interventional or treatment arms receives the DPSCs infusions at days 1, 3, and 7	Primary outcome measure includes the disappear time (calculated by Kaplan-Meier method) of ground-glass shadow in the lungs (time frame: 14 days). Secondary outcome measures include: (1) Lung shadow absorption (Kaplan-Meier method) by CT scan-chest (time frame: 7, 14, 28, and 360 days; (2) Changes of blood oxygen (blood oxygen values compared by *t*-test) (time frame: 3, 7, and 14 days	Not yet recruiting. NCT04302519. https://clinicaltrials.gov/ct2/show/NCT04302519
A case report	One patient underwent sinus lift elevation with DPSCs micro-grafts gentle poured onto collagen sponge	A CT scan control was performed after 4 months and DICOM data were processed with medical imaging software to extract the bone density. Pearson’s Chi-square test was used to investigate difference in bone density between native and the newly formed bone. Bone density in newly formed bone was about twice of native bone, indicating that micro-grafts derived from DPSCs poured onto collagen sponge were a useful method for bone regeneration in atrophic maxilla	Brunelli et al., 2013
A case report	This study assessed the clinical and radiographic regenerative potential of autologous DPSCs in treating human non-contained intraosseous defects	A chronic periodontitis patient requiring extraction of the 3^rd^ molar underwent surgery for extraction of the affected molar. Autologous DPSCs were used to regenerate the infra-bony defect on the mandibular right second premolar. At 1-year follow-up examination, the defect was completely filled with bonelike tissue as confirmed by the reentry procedure	Aimetti et al., 2014
A randomized controlled clinical trial	This study evaluated whether the DPSCs delivered into intra-bony defects in a collagen scaffold would ameliorate the clinical and radiographic parameters of periodontal regeneration	Twenty-nine chronic periodontitis patients requiring extraction of one vital tooth were consecutively enrolled. Defects were randomly assigned to test (autologous DPSCs micrografts seeded onto collagen sponge) or control treatments (collagen sponge alone). Clinical and radiographic parameters were recorded at baseline, 6 and 12 months postoperatively. This study concluded that the application of DPSCs significantly improved clinical parameters of periodontal regeneration at 1 year post-treatment	Ferrarotti et al., 2018
A case report	This clinical trial reports the preliminary findings in a patient with periodontal disease who was successfully grafted with allogeneic DPSCs	DPSCs were passaged and cultured without supplementation and 5 × 10^6^ allogeneic DPSCs in 250 μL PBS were seeded onto a dry scaffold of lyophilized collagen-polyvinylpyrrolidone sponge placed in the left lower premolar area of a 61-year-old patient with periodontal disease. At 3- and 6-months follow-ups, there was no sign of rejection, with reduced tooth mobility, periodontal pocket depth and bone defect area but increased bone mineral density at the graft site, suggesting that DPSCs allograft was a promising treatment for correcting bone defects induced by periodontal disease	Hernández-Monjaraz et al., 2018
A single-center, double-blind, randomized, split-mouth, controlled clinical trial	This clinical trial of 32 patients tested the efficacy of autologous DPSCs delivered in a collagen matrix for post-extraction socket healing	Both impacted mandibular 3rd molars were extracted and resulting DPSCs seeded on a resorbable collagen matrix were implanted in 32 experimental post-extraction sockets, whereas collagen matrices alone were implanted in 32 contralateral control post-extraction sockets. At 6 months post-extraction, CT and an advanced display platform was used to record extraction socket density and compared with measurements obtained immediately after extraction. However, the investigators were unable to show that autologous DPSCs reduce socket bone resorption after inferior 3rd molar extraction	Barbier et al., 2018

Of note, owing to their anti-inflammatory properties and regenerative potentials, DPSCs are also being tested for their therapeutic benefits in coronavirus disease 2019 (COVID-19) patients. Like in acute respiratory distress syndrome (ARDS), COVID-19 patients show loss of alveolar structures and invasion/accumulation of proinflammatory M1 macrophages, resulting in the release of proinflammatory cytokines/mediators and enhanced tissue fibrosis. Interestingly, Wakayama et al. (2015) demonstrated earlier in a mouse model study of acute lung injury that intravenous infusion of DPSC/SHED or the conditioned media potentiated the anti-inflammatory effects via M2 macrophage activation and ameliorated the disease pathophysiology. Currently, few clinical trials are in progress to test out the safety and efficacy of DPSCs-based therapies in COVID-19 patients (Table 4).

## Concluding Remarks and Future Prospectives

Dental pulp is a promising source of DPSCs, which are multipotent stem cells with potentials of self-renewal, multilineage differentiation, and immunomodulatory functions. These stem cells offer the advantage of more comprehensive clinical applications as compared to MSCs derived from other sources like the peripheral blood, adipose tissue, umbilical cord, and bone marrow. Other intriguing aspects that make the use of DPSCs more attractive is their easy access from the discarded third molar tooth and the minimal ethical concerns are involved in the procurement process, as well as the fact that cryopreserved DPSCs will retain their ability for multilineage differentiation into osteogenic, chondrogenic, dentinogenic, myogenic, neurogenic, and adipogenic lineages. Not surprisingly, DPSCs-based stem cell therapy approaches are currently being exhaustively investigated.

However, it is noteworthy that dental pulp has the element of heterogeneity involved as it is a mixture of different cell types that can differentiate into multiple lineages. Of note, first, dental pulp-derived single-cell suspensions need to be cultured to allow the development of individual clones. Next, individual clones are isolated and single cell types are enriched by extended culture expansion, followed by immunophenotyping to characterize the subpopulations based on molecular and phenotypic markers that regulate their differentiation potential into multiple lineages using either 2- or 3-dimension culture conditions in defined media which promote the desired cell lineage specification (Al Madhoun et al., 2016, 2018). Although, fluorescence-activated cell sorting techniques are applicable and improve single-cell population purity, these approaches have several limitations including the exposure of cells to electric charge which may alter their integrity (Tirino et al., 2011). DPSCs’ capacities to differentiate into various lineages are driven by donor age, genetics, and epigenetic factors such as growth and differentiation factors and culture settings used (Zhou D. et al., 2020). Indeed, further research toward standardization of DPSCs’ isolation and culture protocols is still needed. There is also a pressing need for identifying the markers that more specifically and consistently represent DPSCs. Moreover, it is speculated that the identification of such markers will facilitate the direct purification techniques which will minimize DPSCs’ exposure to culture conditions needed for their prospective medical applications. Regarding cell-banking aspects, new cryopreservation media and optimized methods may have to be established to maintain the viability and immunobiological characteristics of the DPSCs over long term use. Similarly, further studies will also be required to better understand the molecular mechanisms that regulate interactions between DPSCs and various biomaterials. The studies employing transmission electron microscopy may help characterizing different phenotypes of the heterogeneous progenitor cells that populate dental stem cell niches and a rigorous testing of the endothelial-mesenchymal transformation will be required to assess their potential of replenishing these niches.

Last but not least, DPSCs-related cellular or secretome based therapeutic interventions used in pre-clinical and clinical trials have yielded promising outcomes. However, there is a growing need for conducting more clinical trials to further establish the safety and efficacy of the DPSCs-based interventions as a powerful therapeutic tool and to lead development in regenerative medicine. Not surprisingly, major challenges still remain before the DPSCs-based interventions can be translated into clinical application to patients (Yamada et al., 2019). Nevertheless, innovated procedures have been used to develop immortalized DPSCs such as mutant baculovirus-based piggyBac system (Li et al., 2020), and DPSCs transduction with CDK4^R24C^ (Orimoto et al., 2020), cyclin D1 or telomerase reverse transcriptase (Wilson et al., 2015). Furthermore, CRISPR gene editing technology has been recently applied to study the functional role of genetic variations in patient-derived DPSCs such as the role of TRPV4 polymorphism (c.1855C > T), a gene known to be implicated in metatropic dysplasia disease (Kang et al., 2012; Nonaka et al., 2019). Nonaka et al. (2019) studies revealed that this gain of function mutation is associated with alternations in calcium/NFATc1 signaling pathway, which in turn accelerates chondrogenic and osteogenic differentiation of DPSCs causing the congenital skeletal disease (Han et al., 2020). Taken together, these outstanding approaches highlight the importance of DPSCS as an adult stem cell model for prospective contribution in the future development of treatment strategies for human diseases.

## Author Contributions

AA, SS, and RA wrote first draft of the manuscript. DH prepared the figures and tables. DH, MA, and FA-M were involved in discussing, drafting, and editing the manuscript. All authors contributed to the drafting and critical review of the manuscript and approved the final draft.

## Conflict of Interest

MA is employed by Biointelligence Technology Systems S.L., Barcelona, Spain. The remaining authors declare that the research was conducted in the absence of any commercial or financial relationships that could be construed as a potential conflict of interest.

## Publisher’s Note

All claims expressed in this article are solely those of the authors and do not necessarily represent those of their affiliated organizations, or those of the publisher, the editors and the reviewers. Any product that may be evaluated in this article, or claim that may be made by its manufacturer, is not guaranteed or endorsed by the publisher.

## Abbreviations

HGF: hepatocyte growth factor
EGF: epidermal growth factor
VEGF: vascular endothelial growth factor
bFGF: basic fibroblast growth factor
PDGF: platelet-derived growth factor
dex: dexamethasone
ITS: insulin-transferrin-selenium
ITS-X: insulin-transferrin-selenium-ethanolamine
LDL: low density lipoprotein
FGF-4: fibroblast growth factor-4
HNF-4: nuclear factor-4 alpha
IFG-1: insulin-like growth factor-1
TAT: tyrosine amino transferase
KOSR: knock-out serum replacement
GLP-1: glucagon-like peptide
NEAAs: non-essential amino acids;.
PHA: phytohemagglutinin
MLR: mixed lymphocyte reaction
LPS: lipopolysaccharide
PBMC: peripheral blood mononuclear cell
Mdr-1: multidrug resistance protein-I
cyp-2e1: cytochrome P450-2E1
Epo: erythropoietin
Pdx-1: pancreas/duodenum homeobox protein 1
Hhex: homeobox protein HEX
MNX1: motor neuron and pancreas homeobox protein 1
Pax-4/-6: paired box protein Pax-4 and 6
NKX6-1: homeobox protein Nkx-6.1
NDNF: neuron-derived neurotrophic factor
NT-3: neurotrophin-3
NGF: beta-nerve growth factor
GDNF: glial cell line-derived neurotrophic factor
FoxA1: forkhead box A1
HNF-3- α: hepatocyte nuclear factor 3-alpha
FoxA2: forkhead box A2
HNF-3- β: hepatocyte nuclear factor 3-beta
GLUT-2: glucose transporter-2
OSM: oncostatin M
β-ME: beta-mercaptoethanol
AMY2A: pancreatic alpha-amylase-PA
Act A: activin A.
